# Numerical and Theoretical Study of Tunable Plasmonically Induced Transparency Effect Based on Bright–Dark Mode Coupling in Graphene Metasurface

**DOI:** 10.3390/nano10020232

**Published:** 2020-01-29

**Authors:** Qichang Ma, Jianan Dai, Aiping Luo, Weiyi Hong

**Affiliations:** Guangzhou Key Laboratory for Special Fiber Photonic Devices and Applications & Guangdong Provincial Key Laboratory of Nanophotonic Functional Materials and Devices, South China Normal University, Guangzhou 510006, China; qichangma@m.scnu.edu.cn (Q.M.); 2018022033@m.scnu.edu.cn (J.D.); luoaiping@scnu.edu.cn (A.L.)

**Keywords:** metasurface, mid-infrared region, graphene split-ring resonators, graphene cut wire resonator, plasmonically induced transparency effect

## Abstract

In this paper, we numerically and theoretically study the tunable plasmonically induced transparency (PIT) effect based on the graphene metasurface structure consisting of a graphene cut wire (CW) resonator and double split-ring resonators (SRRs) in the middle infrared region (MIR). Both the theoretical calculations according to the coupled harmonic oscillator model and simulation results indicate that the realization of the PIT effect significantly depends on the coupling distance and the coupling strength between the CW resonator and SRRs. In addition, the geometrical parameters of the CW resonator and the number of the graphene layers can alter the optical response of the graphene structure. Particularly, compared with the metal-based metamaterial, the PIT effect realized in the proposed metasurface can be flexibly modulated without adding other actively controlled materials and reconstructing the structure by taking advantage of the tunable complex surface conductivity of the graphene. These results could find significant applications in ultrafast variable optical attenuators, sensors and slow light devices.

## 1. Introduction

Typical electromagnetically induced transparency (EIT) occurs in a coherently driven atomic system, resulting from the destructive interference of two dressed states, and a narrow transparency window is generated simultaneously in a broad absorption spectral region [1,2,3]. However, the realization of the traditional quantum EIT requires harsh and unique conditions, namely, stable optical pumping and a cryogenic temperature, which quite constrains further investigations and practical applications [2]. To overcome these barriers, EIT was introduced to metamaterial structures, and the plasmonic analogue of EIT, or PIT (plasmonically induced transparency), based on metamaterial has received significant interest in many fields for its flexible design, no pumping required, room temperature, and easy realization [4,5,6]. Furthermore, the PIT phenomenon has promise for many practical applications, such as high-sensitivity sensors [7], polarization conversions [8], and enhanced nonlinear effects [9]. However, most studies focusing on the metallic nanostructure and the performance of the metallic structure will be constrained for the reason that the permittivity of the metal is difficult to be modulated and its material losses are enormous. Particularly, once the metamaterial structures are fabricated, the spectral response and operating frequency will be fixed, which greatly limits its modulation range and scope of application. In order to implement the dynamically adjustable function of the metamaterial structure, it is essential to reconstruct the structural geometries, which is very impractical and difficult once the devices are fabricated. 

So far, several optical active materials such as semiconductors [10,11] and nonlinear media [12] are integrated into the metallic metamaterial to carry out the dynamic tunability of the PIT window. Recently, graphene, a new two-dimensional material, appears to be a significant candidate to achieve tunable characteristics of metamaterial in the terahertz region since it exhibits many unique and fantastic physical properties such as stable optical response [13], low propagation losses [14], and ultra-high electronic mobility [15]. Particularly, it is found that the complex surface conductivity of the graphene can be flexibly controlled with different Fermi levels by utilizing external electrical gating or chemical doping according to the Kubo equation [16]. Yuan et al. realized the enhancement of absorption of the metasurface with graphene cut wires [17]. Fan et al. made the tunability of the Goos–Hänchen effect is realized by utilizing a graphene metasurface [18]. Tasolamprou et al. experimentally fabricated a tunable absorber based on a graphene metasurface, which can be modulated on the picosecond level [19]. The above studies verified the importance of the graphene in dynamically active metamaterials. However, it is found that they mostly focus on the THz region and the unit cell of the structure is in the order of a micrometer. By contrast, we introduced the graphene into the proposed metasurface whose unit cell is in the order of a nanometer, and we mainly investigated the tunable PIT effect in the mid-infrared region.

In this paper, we propose a tunable metasurface structure to theoretically and numerically study the PIT effect, which consists of a graphene cut wire (CW) resonator and split-ring resonators (SRRs). It is found that a narrow transparency window with 90% transmission appears at 6.08 µm in the transmission spectra due to the weak hybridization between the graphene CW resonator and SRRs. We theoretically calculate the transmission spectra with various coupling coefficients according to the coupled harmonic oscillator model, and the results are approximately consistent with the numerical simulation results from different coupling distances, achieved by utilizing the finite-difference time-domain (FDTD) solutions software. Moreover, the optical response of the proposed metasurface structure can be tuned by controlling structural geometries such as the coupling distance between the graphene SRRs and CW resonator, the number of the graphene layers and the geometries parameters of the graphene CW resonator. Importantly, compared with the metal-based metamaterials, the tunable transparency window can be realized by utilizing the electrical gating to alter the Fermi level of the graphene without adding other actively controlled materials and reconstructing the proposed structure. The results will open a new avenue in tunable sensors, ultrafast optical switches and variable optical attenuators.

## 2. Materials and Methods 

**Figure 1:**
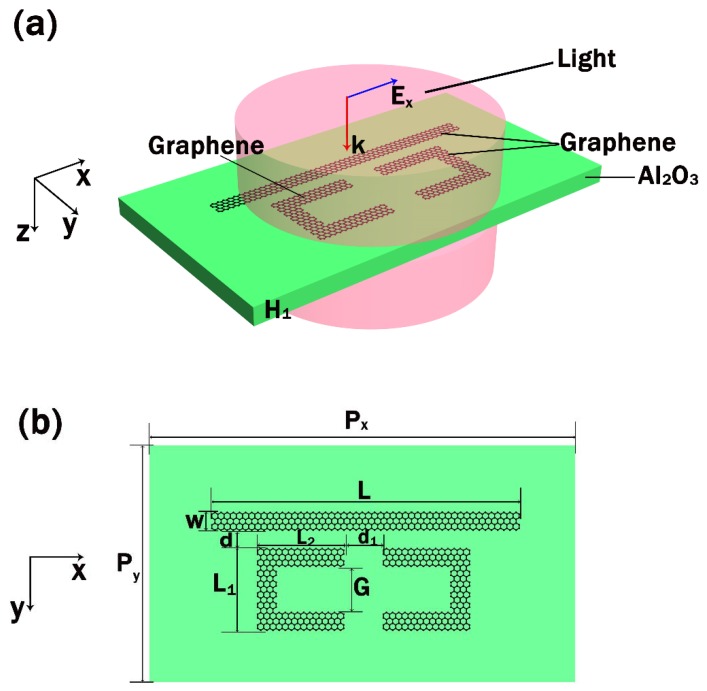
(**a**) Schematic of the unit cell of proposed graphene metasurface composed of graphene cut wire (CW) resonator and split-ring resonators (SRRs). (**b**) Top view of the unit cell with the geometrical parameter $P_{x}=125\;\mathrm{nm}$, $P_{y}=66\;\mathrm{nm}$, L=85 nm, W=7 nm, d=6 nm, $L_{1}==29\;\mathrm{nm}$, $L_{2}=25\;\mathrm{nm}$, $d_{1}=5\;\mathrm{nm}$, G=19 nm and the thickness of the substrate is $H_{1}=40\;\mathrm{nm}$.

The unit cell of the proposed metasurface structure used to investigate the PIT effect is composed of a graphene CW resonator and two identical and axisymmetric graphene SRRs on the Al_2_O_3_ substrate as illustrated in Figure 1a,b. In the simulation, the x-polarized incident plane wave propagates perpendicularly into the graphene metasurface, and the geometric parameters of the structure are provided in the caption. That is to say, the incident light is s-polarized. The thickness of the Al_2_O_3_ substrate whose complex refractive index can be obtained in [20] is set as 40 nm, while the length and width of the graphene CW resonator are *L* = 85 nm and *W* = 7 nm, respectively. The distance of the SRRs is *d_1_* = 5 nm, while the coupling distance between the CW resonator and SRRs is *d* = 6 nm. *L*_1_, *L*_2_ and *G* refer to the length in the y and x-direction and the gap of the split-ring resonator and their detail values are set as *L*_1_ = 29 nm, *L*_2_ = 25 nm, and *G* = 19 nm, respectively. The period of the proposed metasurface structure is *P_x_* = 125 nm in the x-direction and *P_y_* = 66 nm in the y-direction.

The proposed graphene-based metasurface can be fabricated in the following sequence of steps. First of all, the substrate Al_2_O_3_ with the thickness of 40 nm can be grown by atomic layer deposition [21]. Next, the graphene layer can be fabricated via the chemical vapor deposition (CVD) method and the hydrogen-etching method on the copper foil [22,23]. Finally, transfer the above graphene layer onto the Al_2_O_3_ substrate by the dry or wet transfer method [24]. The proposed graphene metasurface with SRRs and the CW resonator can be manufactured after completing the above fabrication process.

The surface conductivity of monolayer graphene σ(ω) consists of intraband and interband transitions, which can be calculated within the random-phase approximation as [25]:

(1) $$\sigma \left(\omega \right)=\sigma_{\mathrm{int}raband}+\sigma_{\mathrm{int}erband},$$

where:

(2) $$\sigma_{\mathrm{int}raband}=\frac{{2\mathrm{ie}}^{2}k_{B}T}{\pi \hbar^{2}\left(\omega +i\tau^{-1}\right)}\ln{\left(2\cosh\left(\frac{E_{f}}{2k_{B}T}\right)\right)}_{,}$$

(3) $$\sigma_{\mathrm{int}erband}=\frac{e^{2}}{4\hbar }{\left(\frac{1}{2}+\frac{1}{\pi }\arctan\left(\frac{\hbar \omega -2E_{f}}{2k_{B}T}\right)-\frac{i}{2\pi }\ln\left(\frac{{\left(\hbar \omega +2E_{f}\right)}^{2}}{{\left(\hbar \omega -2E_{f}\right)}^{2}+{\left(2k_{B}T\right)}^{2}}\right)\right)}_{,}$$

Here, T is the ambient temperature (300 K in this paper), while ω and ℏ refer to the angular frequency of the incident plane wave and the reduced Planck’s constant, respectively. $E_{f}$ expresses the Fermi level and $k_{B}$ is the Boltzmann constant, e and τ refer to the charge of an electron and the carrier relaxation time, respectively. τ can be calculated by the formula τ=1/2Γ [26], where is the scattering rate of the graphene and its value is 0.00099 eV in the simulation, and the corresponding τ is about 0.33 ps. From the above formulas, we can see that the complex surface conductivity of the graphene is especially related to $E_{f}$, and $E_{f}$ is proportional to the Fermi velocity $V_{f}$ and $\sqrt{n}$ according to formulas $E_{f}=\hbar V_{f}{\left(\pi n\right)}^{\frac{1}{2}}$ and $n=\varepsilon_{0}\varepsilon_{d}V_{g}/eH_{1}$ [27,28]. Here, $n,\varepsilon_{0},\varepsilon_{d},V_{g},H_{1}$ are the carrier density, the permittivity in vacuum, the permittivity of Al_2_O_3_, the external voltage on graphene and the thickness of Al_2_O_3_, respectively. We employ the $V_{f}=10^{6}m/s$ [27] and $H_{1}=40\;\mathrm{nm}$ in this paper. The in-plane permittivity of the graphene can be estimated by the following formula for the reason that graphene is equivalent to a thin anisotropic bulk material in the numerical simulations and theoretical calculations [25]:

(4) $$\varepsilon =1+{\frac{i\sigma \left(\omega \right)}{\varepsilon_{0}\omega t_{G}}}_{,}$$

where $t_{G}$ refers to the thickness of the monolayer graphene and its value equals 1 nm in the simulation, while $\varepsilon_{0}$ expresses the permittivity in vacuum [29]. Particularly, to enhance the modulation depth of the transmission spectra, the number of the graphene layers of the CW resonator and SRRs is set as 5 [30], and the conductivity of the N-layer graphene is Nσ (N≤6) [31,32].

In this paper, the FDTD solutions software is utilized to numerically study the optical response of the metasurface structure, and the periodic boundary condition is employed in the x and y directions, while the perfectly matched layer (PML) absorbing boundary condition is applied along the propagation direction of the incident light.

## 3. Results and Discussion

**Figure 2:**
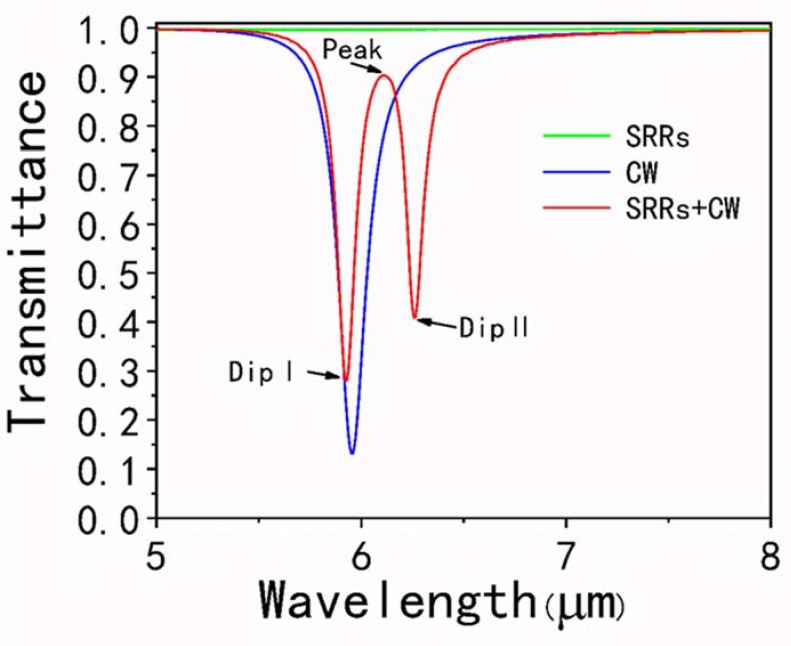
Transmission spectra of the individual graphene CW resonator, the individual graphene SRRs and the plasmonically induced transparency (PIT) metasurface consisting of the two resonators under x-polarized incident light excitation, respectively.

As shown in Figure 2, the transmission spectra of three kinds of the unit cell, one with individual graphene SRRs, one with an individual CW resonator, and one with both of them, are numerically calculated under the x-polarized incident plane wave to clarify the underlying mechanism of the PIT effect. One can see that the incident plane wave directly couples to the graphene CW resonator and a traditional localized surface plasmon (LSP) resonance can be observed at the wavelength around 5.95 μm in the transmission spectra as demonstrated by the solid blue line. By contrast, the graphene SRRs are difficult to be directly excited by the incident plane wave, and the transmission rate is approximately 100% from 5 μm to 8 μm as illustrated with the solid green line. Thus, the graphene CW resonator and SRRs are treated as the bright and dark mode resonator, respectively. When we place the graphene CW resonator and graphene SRRs together, a sharp and narrow PIT window located between two obvious dips at 5.93 μm and 6.26 μm can be observed, with transmittance of 90% at the wavelength around 6.08 μm due to the destructive coupling between bright and dark mode, which is indicated with the solid red curve. In the simulation, the Fermi level of the graphene is $E_{f}=0.9\;\mathrm{eV}$ while the coupling distance between the CW resonator and SRRs is d=6 nm.

**Figure 3:**
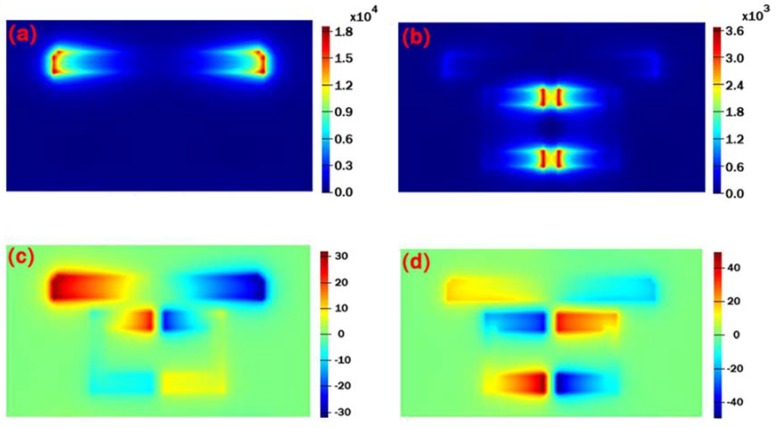
The simulated electric field of |E_z_|^2^-component of (**a**) the individual graphene CW resonator at 5.95 μm and (**b**) the PIT metasurface with CW resonator and SRRs at 6.08 μm. The E_z_ field distribution of the PIT metasurface with graphene CW resonator and SRRs at (**c**) 5.93 μm and (**d**) 6.26 μm.

Then, we numerically calculated the electric field of the |E_z_|^2^-component of the unit cell with individual graphene CW resonator at 5.95 μm and the PIT metasurface at 6.08 μm to explore intensively the physical mechanism of the PIT effect as shown in Figure 3a–d. The power monitor is placed at 2 nm above the graphene layer. One can see that the incident plane wave directly couples to the individual graphene CW resonator and the electric field primarily localizes on the edges and corners as depicted in Figure 3a, which confirms that the graphene CW resonator is acting as the bright mode. By contrast, it is worth noting that the electric field on the graphene CW resonator almost vanishes and it mainly concentrates on the clearance of the two SRRs when the graphene CW resonator and SRRs are integrated on the Al_2_O_3_ substrate as illustrated in Figure 3b, which is the feature of the typical EIT phenomenon [33]. In this configuration, it is found that the strong electric field on graphene CW resonator is transferred to the clearance of the graphene SRRs with the change of the unit cell of the structure, and a sharp and narrow PIT window appears simultaneously on the transmission spectrum due to the destructive interference between the bright and dark mode. 

In addition, the Ez field distribution of the graphene SRRs and CW resonator at two transmissions—dip I (λ=5.93 μm) and dip II (λ=6.26 μm)—is numerically calculated as illustrated in Figure 3c,d. As shown in Figure 3c, the electric field primarily localizes on the CW resonator, which indicates that the incident plane wave couples to the CW resonator strongly, while SRRs are excited weakly at a wavelength of λ=5.93 μm. When it comes to the condition at the wavelength of λ=6.26 μm, the light field conversely concentrates on the SRRs while the CW resonator is hardly excited. It reveals that dip I is mainly dominated by the resonance of graphene CW resonator, while dip II is dependent on the resonance of the graphene SRRs. In the simulation, the coupling distance d is set as 6 nm, and the Fermi level $E_{f}$ is fixed as 0.9 eV, while the other geometrical parameters of the structure remain unchanged.

**Figure 4:**
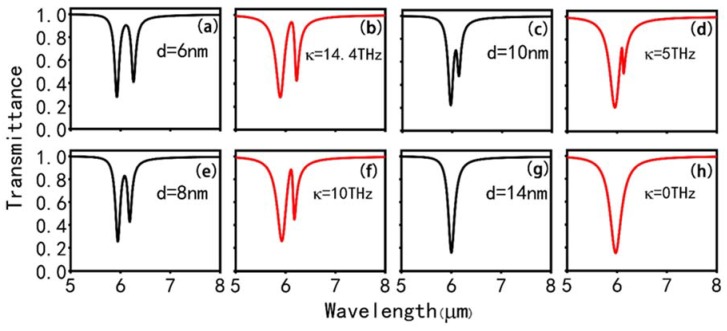
(**a**,**c**,**e**,**g**) the simulated transmission spectra of the PIT metasurface with different coupling distances *d*. (**b**,**d**,**f**,**h**) corresponding theoretical transmission spectra with different coupling coefficients *κ*.

Successively, we study the dependence of the PIT effect on the coupling distance d between the CW resonator and SRRs. The transmission peak gradually grows sharper, and its transmittance becomes lower with the increase of d, as depicted in Figure 4a,c,e,g. Furthermore, one can see that the transmission dip I is red-shift while the dip II is blue-shift when d increases from 6 to 10 nm. Simultaneously, the dip II ascends while the dip I gradually drops with the increase of d. Moreover, there is only one transmission dip at 6 μm, and the PIT phenomenon disappears when d comes to 14 nm. 

To get further insight into the above behaviors of the PIT effect, the coupled harmonic oscillator model, similar to the three-level atomic system, is adopted to make the quantitative analysis on the coupling between the CW resonator and SRRs, which contains a ground state |0〉 and two excited states |1〉 and |2〉. The dipole-allowed transition is expressed as |0〉-|1〉 and it is analogue to the bright mode resonance in a graphene CW resonator, while |0〉-|2〉 expresses the dipole-forbidden transition, corresponding to the dark mode resonance in SRRs. Consequently, it gives rise to the destructive interference of two possible channels: |0〉-|1〉 and |0〉-|1〉-|2〉-|1〉, which suppresses the losses and enhances the transmission [3]. The interference of two possible channels can be nicely calculated by utilizing coupled differential equations [33,34]:

(5) $${\ddot{x}}_{1}+\gamma_{1}{\dot{x}}_{1}+\omega_{0}^{2}x_{1}+\kappa x_{2}=E_{,}$$

(6) $${\ddot{x}}_{2}+\gamma_{2}{\dot{x}}_{2}+{\left(\omega_{0}^{}+\delta \right)}^{2}x_{2}+\kappa x_{1}=0_{,}$$

where $x_{1}$, $x_{2}$, $\gamma_{1}$, $\gamma_{2}$ refer to the amplitudes and damping rates of the bright and dark mode, respectively. $\omega_{0}$ expresses the resonance angular frequency of the metasurface structure with an individual graphene CW resonator, while $\omega_{0}+\delta$ denotes the resonance frequency of the dark mode. E and κ refer to the incident electromagnetic field and coupling coefficient between the graphene CW resonator and SRRs, respectively. After solving Equations (5) and (6) with the approximation $\omega -\omega_{0}\ll \omega_{0}$, the susceptibility can be calculated as [35,36]:

(7) $$x=x_{r}+ix_{i}\propto {\frac{\left(\omega -\omega_{0}^{}-\delta \right)+i\frac{\gamma_{2}}{2}}{\left(\omega -\omega_{0}^{}+i\frac{\gamma_{1}}{2}\right)\left(\omega -\omega_{0}^{}-\delta +i\frac{\gamma_{2}}{2}\right)-\frac{\kappa^{2}}{4}}}_{,}$$

The transmission T can be calculated as $T=1-gx_{i}$ for the reason that $x_{i}$ is related to energy dissipation, where the geometric parameter g denotes the coupling strength between the bright mode resonator and E.

**Figure 5:**
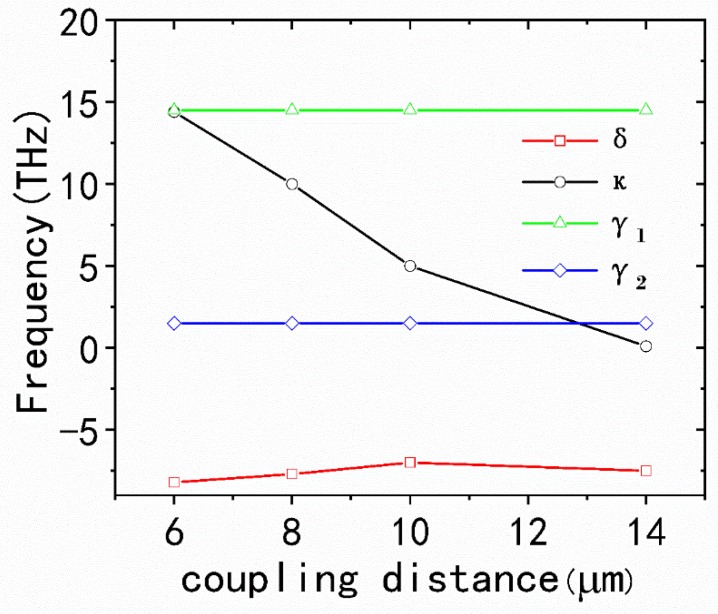
The fitting parameters $\gamma_{1}$, $\gamma_{2}$, δ and κ as a function of the coupling distance between the graphene CW resonator and graphene SRRs.

Figure 4b,d,f,h shows the analytical fitted transmission spectra with different coupling coefficients κ corresponding to the transmission spectra with different coupling distances d and the theoretical results are approximately consistent with the numerical simulation. Moreover, the value of the fitting parameter with increasing coupling distance can be obtained in Figure 5. It can be nicely observed that the $\gamma_{1}$, $\gamma_{2}$, δ are almost unchanged, while the coupling coefficient κ drops from 14.4 THz to 0 THz when d ascends from 6 to 14 nm for the x-polarized incident plane wave. As shown in Figure 5, it is found that the transmission spectra greatly depends on the κ, and when the κ approaches 0, the PIT effect disappears and only an LSP resonance remains at λ=6.0 μm.

**Figure 6:**
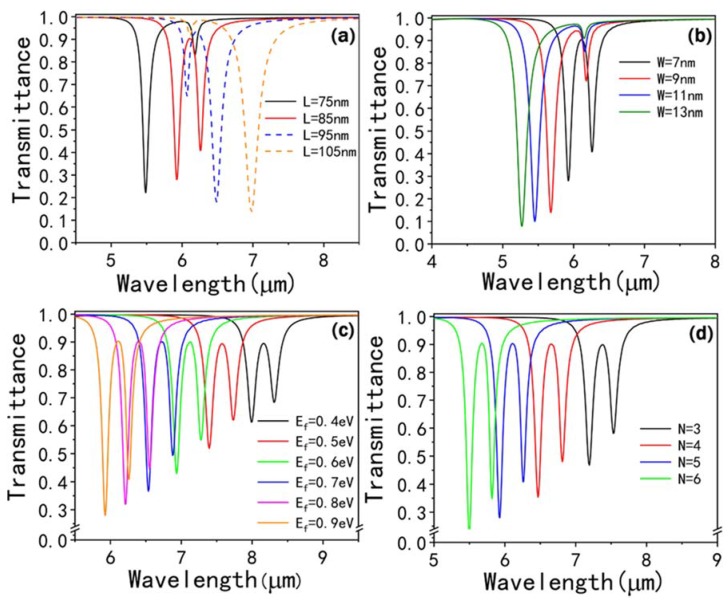
The transmission spectra of the PIT metasurface with different (**a**) L, (**b**) W, (**c**) *E_f_* and (**d**) N.

Then, we explore the influence of the geometrical parameters of the graphene CW resonator on the PIT spectra. Figure 6a depicts the transmission spectra with different L, and it is found that both the dip I and dip II have a red-shift with the increasing L. In addition, when L increases from 75 to 85 nm, the transmission peak and dip II become distinct, while the modulation depth of dip I decreases slightly. Moreover, once the L is greater than 85 nm, the transmission peak gradually vanishes with the increasing L, and the modulation depth of dip I becomes lower than dip II. When L approaches 105 nm, a distinct plasmon resonance at 7 μm can be observed, and dip I almost disappears. In Figure 6b, the width W of the graphene CW resonator can also influence the PIT phenomenon. The transmittance of dip II drops rapidly from 91% to 41% with the decreasing W. When the W increases from 7 to 13 nm, both dip I and dip II have a blue-shift, and an obvious PIT phenomenon can be observed when W approaches 7 nm. 

As mentioned above, the complex surface conductivity of the graphene can be flexibly controlled by $E_{f}$ via utilizing gating voltage without adding other actively controlled materials or reconstructing the proposed structure, which is one of the principal advantages of the graphene metasurface compared to metal-based metamaterial. Although the proposed graphene metasurface composed of graphene CW resonator and SRRs is disconnected, a covering of ion-gel can be employed to apply gating voltage [37,38,39]. The ion-gel gate dielectric materials are transparent and possess the characteristics of excellent electrochemical and thermal stability and significant mechanical flexibility [40,41,42]. Particularly, it has limited effects on graphene plasmon lifetimes, which make it a good candidate for tunable graphene metasurface [43].

In the simulation, the $E_{f}$ of graphene CW resonator and SRRs change simultaneously and their value is the same. When $E_{f}$ increases from 0.4 eV to 0.9 eV, both dip I and dip II have a blue-shift and their transmission rate drops most noticeably, as depicted in Figure 6c. One can see that the transmission peak exhibits a blue-shift from 8.17 μm to 6.08 μm, and its transmission rate is approximately the same with the increasing Fermi level. The above behaviors can be interpreted by the formula $\lambda_{r}\propto \sqrt{2\pi^{2}\hbar cW/\left(\alpha_{0}E_{f}\right)}$, where $\lambda_{r}$ and W refer to the resonant wavelength and the width of the graphene CW resonator, respectively [44]. $\alpha_{0}=e^{2}/\left(\hbar c\right)$ expresses the fine structure constant. Consequently, $\lambda_{r}\propto \sqrt{1/E_{f}}$ for the reason that the width of the graphene CW resonator is fixed as 7 nm in the simulations and other variables remain unchanged, which significantly validates the simulation results. In addition, we investigate the evolution of transmission spectra with different numbers of graphene layers on the PIT spectra as shown in Figure 6d. One can see that the transmission dips and peak are blue-shifted when the N increases from 3 to 6. Moreover, the transmission rate of the resonance peak is approximately unchanged with the increasing N and the resonance strength of a transmission dip becomes stronger. 

## 4. Conclusions

We have numerically and theoretically studied the tunable PIT effect of the proposed metasurface in the middle infrared region (MIR), which consists of a graphene CW resonator and SRRs. After optimizing the geometry of the structure, a transmission peak of 6.08 μm with 90% transmission rate appears due to the destructive coupling between the graphene CW resonator and SRRs for the x-polarized incident light. The electric field distributions are calculated to further investigate the physical mechanism of the PIT effect. In addition, it is found that the transmission spectra of the proposed metasurface particularly depends on the geometrical dimensions such as the geometrical parameters of the graphene CW resonator, the number of graphene layers, and the coupling distance. The coupled harmonic oscillator model is employed to make the quantitative analysis on the situation with different coupling distances, which is approximately consistent with the numerical simulations. Particularly, compared with metal-based metamaterials, the proposed graphene metasurface can achieve the dynamically tunable PIT window in a wavelength range from 6.08 μm to 8.17 μm by altering the $E_{f}$ of the graphene via gating voltage without adding other actively controlled materials and reconstructing the proposed structure. This is contributive to the fabrication of multi-wavelength applicable devices such as the sensors, active modulators and slow light devices in the MIR region.
